# Mini-AFTERc: a controlled pilot trial of a nurse-led psychological intervention for fear of breast cancer recurrence

**DOI:** 10.1186/s40814-023-01431-x

**Published:** 2024-01-08

**Authors:** Calum McHale, Susanne Cruickshank, Tamara Brown, Claire Torrens, Jo Armes, Deborah Fenlon, Elspeth Banks, Tom Kelsey, Gerald Humphris

**Affiliations:** 1 Medical School, North Haugh, University of St Andrews, St Andrews, Fife, KY16 9TF UK; 2 Royal Marsden NHS Foundation Trust, London, UK; 3 Leeds Beckett University, Leeds, UK; 4 NMAHP Research Unit, Stirling, UK; 5 University of Surrey, Guildford, Surrey UK; 6 University of Swansea, Wales, UK; 7 Independent Cancer Patients’ Voice, Carluke, Scotland, UK; 8 Computer Science, North Haugh, University of St Andrews, St Andrews, Fife, KY16 9TF UK

## Abstract

**Objectives:**

To determine the feasibility and acceptability of implementing the Mini-AFTERc intervention.

**Design:**

Non-randomised cluster-controlled pilot trial.

**Setting:**

Four NHS out-patient breast cancer centres in Scotland.

**Participants:**

Ninety-two women who had successfully completed primary treatment for breast cancer were screened for moderate levels of fear of cancer recurrence (FCR). Forty-five were eligible (17 intervention and 28 control) and 34 completed 3-month follow-up (15 intervention and 21 control).

**Intervention:**

Mini-AFTERc, a single brief (30 min) structured telephone discussion with a specialist breast cancer nurse (SBCN) trained to target the antecedents of FCR.

**Outcomes:**

Feasibility and acceptability of Mini-AFTERc and the study design were assessed via recruitment, consent, retention rates, patient outcomes (measured at baseline, 2, 4, and 12 weeks), and post-study interviews with participants and SBCNs, which were guided by Normalisation Process Theory.

**Results:**

Mini-AFTERc was acceptable to patients and SBCNs. SBCNs believe the implementation of Mini-AFTERc to be feasible and an extension of discussions that already happen routinely. SBCNs believe delivery, however, at the scale required would be challenging given current competing demands for their time. Recruitment was impacted by variability in the follow-up practices of cancer centres and COVID-19 lockdown. Consent and follow-up procedures worked well, and retention rates were high.

**Conclusions:**

The study provided invaluable information about the potential challenges and solutions for testing the Mini-AFTERc intervention more widely where limiting high FCR levels is an important goal following recovery from primary breast cancer treatment.

**Trial registration:**

ClinicalTrials.gov, NCT0376382. Registered on 4 December 2018.

**Supplementary Information:**

The online version contains supplementary material available at 10.1186/s40814-023-01431-x.

## Key messages regarding feasibility

1. What uncertainties existed regarding the feasibility?
  Can a training programme for a telephone intervention to prevent fears of cancer recurrence (Mini-AFTERc) designed for specialist breast cancer nurses (SBCNs) be successfully developed?
  Can the intervention be delivered in oncology services and be acceptable to SBCNs?
  Can a formal RCT format be conducted to enable a full trial?
2. What are the key feasibility findings?
  The intervention was acceptable to patients and SBCNs.
  Implementation of Mini-AFTERc was considered feasible by SBCNs.
  Delivery of Mini-AFTERc could vary according to time constraints and different systems of follow-up care.
  Retention in the study was strong.
3. What are the implications of the feasibility findings for the design of the main study?
  Close inspection of SBCN ways of working in each unit is required to understand how to recruit staff and patients.
  Training of staff is a vital element to motivate and standardise recruitment and delivery of this brief intervention.
  Research staff independent of service are required on-site to ensure patient entry and follow-up for the main study.

## Background

Fear of cancer recurrence (FCR) is the ‘fear, worry, or concern relating to the possibility that cancer will come back or progress’ [1] and is a primary concern for cancer survivors. FCR is one of the most reported unmet needs following cancer treatments [2] and is particularly prevalent amongst breast cancer survivors [3]. Persistent and elevated levels of FCR are associated with lower quality of life and poorer mental health outcomes for cancer survivors [4–7]. Furthermore, FCR can increase if not appropriately managed [6]. Psychological support or intervention is necessary to help those affected by FCR to cope with these worries and constructively adapt to life following cancer [6].

Several intensive psychological interventions exist to help support those who experience very high or ‘clinical’ levels of FCR shift towards online appointments and telemedicine in the wake of the COVID-19 pandemic [8, 9] means that delivering such intense FCR interventions at the scale required would be challenging.

The Mini-AFTERc intervention is a structured 30-min telephone discussion that employs cognitive behavioural principles to target and address antecedents of FCR [10–12]. It is designed as a preventative intervention for cancer survivors with elevated or ‘moderate’ levels of FCR. The AFTERc intervention of six face-to-face sessions, developed by Humphris and colleagues, is designed for patients diagnosed with cancer and who have high levels of FCR [13]. Mini-AFTERc differs from AFTERc and other FCR interventions in that it is a brief single-session telephone discussion deliverable by existing members of the cancer care team (e.g. specialist breast care nurses (SBCNs)).

Assessing intervention acceptability and feasibility, as well as piloting study methods, are essential parts of developing and evaluating complex interventions [14, 15]. Preliminary testing of Mini-AFTERc with a small sample of 16 breast cancer survivors suggested the intervention was effective at reducing FCR, and SBCNs who delivered the intervention found that Mini-AFTERc was manageable and simple to follow [10]. Additional survey work by our team with 90 SBCNs across the UK found that current management of FCR was highly variable and Mini-AFTERc could be a consistent and feasible approach, as well as a useful addition to their skillset [16].

The main aim of this pilot study is to investigate the acceptability and feasibility of assessing the Mini-AFTERc intervention in a controlled trial format and of implementing the Mini-AFTERc intervention in everyday clinical practice. Therefore, the objectives of this pilot trial were:

1. To develop a Mini-AFTERc intervention training programme for SBCNs
2. To assess (a) the acceptability of the Mini-AFTERc intervention for SBCNs and patients and (b) the feasibility of introducing the intervention into current practice
3. To assess the feasibility of a controlled trial format and evaluate methodological components, including sample size requirements, to inform the development of a randomised controlled trial

This paper will report on the findings of the Mini-AFTERc pilot trial using the CONSORT statement for pilot and feasibility trials as a reporting framework [17].

## Methods

### Study design

This study used a non-randomised cluster-controlled trial design. A protocol has been published previously [11]. The study took place in four NHS Scotland breast cancer centres. Two centres were intervention centres where participants received the Mini-AFTERc intervention in addition to usual follow-up care. Two centres were control centres where participants received usual follow-up care only. Follow-up practices varied considerably between centres, but no centre had a structured FCR intervention as part of usual follow-up care. Centres were not randomised during this study because SBCNs at one intervention centre had previously received Mini-AFTERc training as part of prior feasibility work [10], and the other intervention centre was aware of the intervention work through GH, who ran a regular psycho-oncology consultant clinic there.

### Participants and sample size

The target population was NHS patients with breast cancer who had recently completed primary cancer treatment within the last 3 months. Patients were eligible to participate if they:

1. Were female
2. Had completed primary cancer treatment for breast cancer within the last 3 months
3. Had no detectable cancer remaining
4. Were ≥ 18 years
5. Their responsible clinician agreed with their participation

Patients who met these criteria also had to score moderately (between 10 and 14) on the Fear of Cancer Recurrence 4-item measure (FCR4) [18] screening questionnaire.

We aimed to recruit 65 participants at intervention centres and 65 at control centres [11]. Sample size calculations indicated that data from 130 participants would be required to demonstrate any effect of the intervention at an effect size of 0.5 and at 0.85 power. This assumed a standard deviation of 7 between *pre-* and *post*-FCR measures (based on previous feasibility work) [10] and an attrition rate of 30%.

### The Mini-AFTERc intervention

Mini-AFTERc is a structured discussion plan, developed to identify how FCR manifests and targets psychological factors known to exacerbate and maintain FCR development [10–12]. Mini-AFTERc is designed to be delivered in a single 30-min telephone call by a SBCN. A previously developed training programme and manual [11] were used to train four SBCNs working at intervention centres to deliver the Mini-AFTERc intervention (two SBCNs at each centre). The Mini-AFTERc discussion covers four key topics associated with FCR formulation and perpetuation, namely:

1. Family
2. Thoughts and feelings
3. Expectations
4. Return of cancer

An initial assessment determines where, within the four key discussion topics, a person may have specific problems. The discussion then focuses on up to two key discussion topics where possible problems are identified. All intervention telephone discussions in this trial were audio recorded so that the fidelity of intervention implementation could be assessed through a tailored measure [19].

### Study procedure

In three of the four participating cancer centres, study information was sent to breast cancer patients ahead of routine post-treatment follow-up clinics. At clinics, clinicians would invite patients to speak with researchers who would answer questions and offer consent forms. As it was not possible to send the information prior to clinics at the remaining centre, the researchers provided study information and consent forms to patients after appointments.

In return for signed informed consent, participants were screened for moderate levels of FCR (defined as scoring 10–14 on the FCR4 measure) [18]. This was conducted in person or over the telephone, depending on how the recruitment process was organised with the cancer centre. Participants who exceeded the FCR4 upper cut-off of 14 were offered psychological support in line with standard practice at each cancer centre. Following the screening, eligible participants completed baseline questionnaires to assess their mood (using the Hospital Anxiety and Depression Scale; HADS) [20] and health-related quality of life (using the EQ-5D questionnaire [21]). Follow-up assessments of the FCR4 and HADS were conducted on three occasions, approximately 2, 4, and 12 weeks following receiving the intervention for participants at intervention centres and following recruitment for patients at control centres. A single follow-up assessment of EQ-5D was also conducted as part of the 12-week follow-up. For follow-up assessments, patients could complete and return paper questionnaires or complete them online using the Mini-AFTERc smartphone app [21]. Researchers digitally coded the paper questionnaires or periodically downloaded the smartphone app data from a secure encrypted server located at the University of St Andrews. Participants recruited at intervention centres received an appointment for the Mini-AFTERc telephone discussion with a trained SBCN within 2 weeks of recruitment. Immediately following the intervention discussion, participants were asked to evaluate their satisfaction with their intervention discussion by completing the Consultation and Relational Empathy (CARE) measure [22] and the Medical Interview Satisfaction Scale (MISS), modified to reflect the Mini-AFTERc discussion [23].

Following trial completion, invites to participate in a post-study semi-structured interview were sent to all participants and SBCNs who delivered the intervention. SC conducted interviews with participants and TB conducted interviews with SBCNs. All interviews were conducted via telephone and were audio recorded for transcription.

### Patient and public involvement

EB is a patient and public representative working with the National Cancer Research Institute and the Independent Cancer Patients’ Voice. EB has been an active and equal partner in all aspects of this study from developing the initial study design through to dissemination of the study findings. EB was consulted about the development of patient documentation, recruitment approaches, and study outcomes. EB regularly contributed to research update meetings during study implementation and to the write-up of this report.

### Study objective 1: develop the Mini-AFTERc intervention training procedure

Development was assessed by how well the planned training programme of three sessions fitted into SBCN working schedules, including the length of sessions, intervals between sessions, and any SBCN dropout. This information was collected from SBCNs following training and during post-study interviews. We also developed a novel measure for assessing the fidelity of the implementation of the Mini-AFTERc intervention [19]. The fidelity measure was developed and tested using transcripts of Mini-AFTERc intervention discussions from an initial feasibility study [10] and from audio recordings of SBCN discussions with a simulated patient as part of the training sessions during this study.

### Study objective 2a: assessing acceptability of the Mini-AFTERc intervention

Normalisation Process Theory (NPT) is a toolkit for understanding and evaluating the implementation and integration of complex interventions [24]. NPT was implemented in this study to assess acceptability and feasibility through post-study semi-structured interviews with participants and SBCNs. The NPT core constructs of coherence (i.e. making sense of the intervention) and cognitive participation (i.e. valuing or ‘buying into’ the intervention) were used to develop and guide questioning about intervention acceptability. Post-study semi-structured interviews asked both participants and SBCNs about their experiences with the Mini-AFTERc intervention. For participants, questioning focused on their understanding of the intervention: how Mini-AFTERc differed from other discussions with SBCNs, perceived benefit and value of Mini-AFTERc, and how Mini-AFTERc impacted their experiences of FCR.

Patient satisfaction measures (CARE and modified MISS) were also collected to assess intervention acceptability for participants who received the intervention, particularly the acceptability of receiving the intervention from SBCNs and the psychosocial focus of the discussion.

### Study objective 2b: assessing feasibility of the Mini-AFTERc intervention

This was explored using the post-study semi-structured interviews with SBCNs and guided by the NTP core constructs of Collective Action (i.e. the work required to introduce new practices or change existing ones) and Reflexive Monitoring (i.e. understanding how new or changed practices affect people). Questioning focused on understanding the intervention, perceptions of value and benefit for participants, how Mini-AFTERc differed from their usual FCR discussions with patients, and whether they would be prepared to continue using Mini-AFTERc in their practice following the study. SBCNs were also asked to discuss factors that may influence the implementation of the Mini-AFTERc intervention, both within a research trial and in routine clinical practice more broadly.

### Study objective 3: assessing trial feasibility and evaluation of methodological components

#### Recruitment and retention

Recruitment data included the number of study information letters sent to patients in addition to the total number of patients who attended recruitment clinics, did not meet the study inclusion criteria, and/or declined to participate. Participant retention after recruitment was assessed by monitoring attendance at intervention discussion sessions and/or returning follow-up assessment questionnaires. The willingness of participants to be randomised in a future trial was also assessed as part of this study. These recruitment data allowed for the calculation of a required sample size for a future RCT.

#### Patient-reported outcomes measures

All participants completed the baseline questionnaires, either in-person (completed the questionnaires themselves) or over the telephone (researchers read out the questions and recorded the patients’ responses). Rates of questionnaire completion and any patterns in missing data were key outcome variables. A secondary outcome variable was to determine any effect of the intervention by examining change in FCR4 scores. During post-study interviews, participants were also asked about the acceptability of the questionnaire measures and the controlled trial study design, including their thoughts on randomisation to intervention or control groups in a future trial.

#### Methodological evaluation

Following the completion of data collection, A Process for Decision-making and Pilot and Feasibility Trials (ADePT) [25] was conducted to assess the overall fidelity of the study’s methodological conduct, including the design and implementation of study procedures. ADePT provided a structured framework for systematically recognising any challenges that occurred during the pilot study and facilitated the identification of potential solutions to these challenges in our planned future work.

### Analysis

Descriptive analysis was conducted on screening and follow-up data in SPSS Version 26 [26]. Comparisons were made between participants recruited at intervention and control centres on demographic variables to identify any key differences between study groups. Missing data analysis was also conducted across the entire dataset to determine the extent of missing data. Little’s Missing Completely At Random (MCAR) test [27] was conducted to determine whether there were any patterns to the missing data.

Psychometric evaluation was performed on the FCR4 measure including Cronbach’s alpha and item-total correlations. Statistical assumptions were also checked including floor and ceiling effects as well as assessments of skewness and kurtosis within the data set.

An intention-to-treat analysis was conducted to determine any effect of the intervention. Self-report FCR data were analysed using mixed linear models with four waves (time) using maximum likelihood estimation controlling for age in years (*mixed* procedure in STATA v15 [28]). Two-sided alpha was 0.05. The intra-class correlation was estimated for future power calculation purposes using the four cancer centres as the clustering factor units.

Normalisation Process Theory [24] provided the framework for the thematic analysis of SBCN and patient interviews. All interviews were transcribed verbatim. Framework analysis was conducted to code transcripts according to the four components of NPT, using NVivo 12.0 [29].

## Results

### Sample overview

Ninety-two patients (41 at intervention centres and 51 at control centres) completed FCR4 screening. Forty-five of these patients (17 intervention and 28 control) met the FCR4 eligibility cutoff [10–14] and participated in the study. Demographic information (collected during screening) for the entire participant sample is presented in Table 1. A larger proportion of participants at control centres were married or in a relationship compared to those at intervention centres (*χ*^2^ (5) = 15.39, *p* < 0.01) and participants screened at intervention centres were more likely to have received surgery (*χ* ^2^ (1) = 5.95, *p* = 0.02) and radiotherapy (*χ*^2^ (1) = 7.83, *p* < 0.01) than participants screened at control centres.

**Table 1:** Demographic details of participants screened

	**Intervention centres**			**Control centres**		
	**Eligible** (*n* = 16)^a^	**Ineligible** (*n* = 24)	**All** (*n* = 40)	**Eligible** (*n* = 28)	**Ineligible** (*n* = 23)	**All** (*n* = 51)
**Age**						
*Mean (SD)*	60.2 (8.3)	56 (9.6)	57.7 (9.2)	57.3 (10.1)	53.6 (10.2)	55.6 (10.3)
**Relationship status** *n* (%)						
Married/partnered	12 (75.0)	11 (45.8)	23 (57.4)	21 (75.0)	21 (91.3)	42 (82.3)
Separated/divorced	3 (18.7)	5 (20.8)	8 (20.0)	2 (7.1)	2 (8.7)	4 (7.8)
Widowed	1 (6.3)	5 (20.8)	6 (15.0)	2 (7.1)	0	2 (3.9)
Single	0	3 (12.5)	3 (7.5)	3 (10.7)	0	3 (5.9)
**Living situation** *n* (%)						
With partner/spouse	12 (75.0)	10 (41.7)	22 (55)	15 (53.6)	14 (60.9)	29 (56.9)
Alone	4 (25.0)	8 (33.3)	12 (30)	6 (21.4)	0	6 (11.8)
With children	0	5 (20.8)	5 (12.5)	0	2 (8.7)	2 (3.9)
Other	0	1 (4.2)	1 (2.5)	7 (25)	7 (30.4)	14 (27.5)
**Education level** *n* (%)						
High school or below	6 (37.5)	6 (25.0)	12 (30.0)	7 (25.0)	3 (13.0)	10 (19.6)
College	6 (37.5)	12 (50.0)	18 (45.0)	14 (50.0)	10 (43.5)	24 (47.1)
University	4 (25)	6 (25.0)	10 (25.0)	7 (25.0)	10 (43.5)	17 (33.3)
**Occupational status** *n* (%)						
Employed	8 (50.0)	17 (70.8)	25 (62.5)	13 (48.2)^b^	18 (78.3)	31 (62.0)
Unemployed	2 (12.5)	0	2 (5.0)	6 (22.2)	3 (13.0)	9 (18.0)
Retired	6 (37.5)	7 (29.2)	13 (32.5)	8 (29.6)	2 (8.7)	10 (20.0)
**Cancer treatments** *n* (%)						
Surgery	16 (100)	24 (100)	40 (100)	22 (78.6)	22 (95.7)	44 (86.3)
Radiotherapy	16 (100)	24 (100)	40 (100)	23 (82.1)	19 (82.6)	42 (82.4)
Chemotherapy	8 (50.0)	11 (45.8)	19 (47.5)	9 (32.1)	8 (34.8)	17 (33.3)
Herceptin^b^	7 (43.8)	30 (12.5)	10 (25.6)	5 (17.9)	1 (4.5)	6 (12.0)

^a^1 patient’s baseline data missing

^b^2 responses missing

#### Study objective 1: develop the Mini-AFTERc intervention training procedure

A three-session Mini-AFTERc training programme was successfully delivered to four SBCNs working in intervention centres. The training content and procedure can be found in the trial protocol paper [11]. Training was delivered by CM, CT, GH, and SC. Each session lasted approximately 3 h and all three sessions were delivered within 1 month at each centre. Four SBCNs received the training, two from each intervention centre. Overall, the training delivery ran smoothly and was considered a success by both researchers and SBCNs. All SBCNs reported to have found the training positive, useful, and relevant for their practice.

#### Study objective 2a: assessing acceptability of the Mini-AFTERc intervention

Two of the 17 intervention participants dropped out before receiving the Mini-AFTERc intervention. CARE and modified MISS measures were successfully completed by 13 of the 15 participants who did receive the intervention. Mean scores for all 10 items in the CARE questionnaire were 4.7 out of a maximum of 5, indicating high levels of intervention acceptability for participants, in terms of emotional and relational empathy provided by SBCNs. Mean modified-MISS scores indicated that participants were neutral about whether talking to the SBCN had relieved their FCR (score 3.4 out of 5) and about their chances of cancer not returning (3.2 out of 5); however, they tended to disagree that talking to the SBCN had not helped with their FCR (1.9 out of 5).

During post-study interviews, patient participants (90% from control centres) expressed a desire for more information at the end of primary treatment, including information about what was normal in terms of new symptoms and guidance about risk reduction, such as lifestyle changes. Overall participants thought that it was important and appropriate to provide psychological interventions for FCR and understood the purpose of the intervention approach taken by the Mini-AFTERc intervention. They believed the Mini-AFTERc intervention was appropriate and a good idea for those who needed it. Few patients thought they themselves needed additional interventions for FCR but were happy to participate because it might “help other people”. Nine of the 10 participants who were successfully interviewed were in the control group and did not receive the intervention. No participant had negative comments, was of an ethical nature, and was entering a future study where randomisation was introduced to place potential participants into control or intervention arms. An intimation was made that control participants be afforded the opportunity to receive the intervention phone call on completion of the final follow-up data point (12 weeks).

SBCNs reported Mini-AFTERc to be a useful and acceptable approach to facilitating conversations about FCR. SBCNs agreed that FCR was a key issue for many of their patients and that soon after the end of primary treatment was the best time to discuss FCR. One SBCN expressed that Mini-AFTERc was very important for the ongoing management of FCR as they felt it formalises what is, typically, an informal discussion. The intervention increases the value and priority of providing planned psychosocial interventions in what was viewed as an increasingly biomedically focused role.

*I found [Mini-AFTERc] incredibly useful, I think more so now the focus has started to be more medical, I think having a tool or having an intervention may make the nurses more aware what goes on and how to deal with it.* SBCN 1

SBCNs valued the structured format of the Mini-AFTERc discussion, which assisted them in managing the content and length of the intervention discussion more confidently and efficiently. For some SBCNs, this also highlighted the lack of structure in many of their routine interactions with patients.

*Confidence but also having a bit more stuctured intervention does help. As breast care nurses you have ideas of what you want to cover in a consultation but I don't think its as structured as an intervention and I think we're very bad at making a consultation open ended.* SBCN 2

#### Study objective 2b: assessing feasibility of implementing the Mini-AFTERc intervention

SBCNs reported that elements of the Mini-AFTERc intervention could feasibly be implemented into their practice and where, in their specific follow-up pathway, they would be best integrated. However, they were not confident that Mini-AFTERc could currently be delivered routinely to all patients coming through the service who expressed concerns about FCR. They indicated that the time required to deliver the intervention, as well as the SBCNs’ already demanding workload, would result in Mini-AFTERc competing with (or being conducted instead of) other necessary work tasks.

*We have a model we use about when we would aim to call people […] we would have to look at that. Is there anything that other people do so we don't need to phone them at that particular time? Certainly for the people that we currently phone, I think it could be incorporated quite well. But could we do it for a thousand people through the unit, probably not.* SBCN 2

There was an evident incongruence in that SBCNs stated that Mini-AFTERc was similar to conversations they conduct with patients on a day-to-day basis, just more formalised and structured; however, they also saw the Mini-AFTERc intervention as being separate from their typical workload and an additional task that they did not necessarily have time to do.

[…] *we took it on as something additional but we didn't have additional time to do it so I would say that if, for example, I was doing one of the calls that would mean a couple of end of treatment calls I would have done that I didn’t do. *SBCN 3

### Objective 3: assessing trial feasibility and methodological components

#### Recruitment and retention

Recruitment started on 13 June 2019. All centres were recruiting on 8 October 2019 with the intention of continuing recruitment until May 2020. Recruitment was prematurely halted on 23 March 2020 due to the COVID-19 pandemic.

In total, 382 patients were identified as potentially eligible (198 in intervention centres and 184 in control centres). Of these, 234 were approached about participating in the study (132 intervention and 102 control) either directly in clinics or over the telephone. The 148 patients who were not approached were found not to meet the inclusion criteria or did not attend their clinic appointments. Subsequently, 96 patients completed study consent (42 intervention and 54 control) and 92 participated in FCR4 screening (41 intervention and 51 control). After the screening, 45 participants met the FCR4 cut-off for inclusion and subsequently enrolled in the pilot trial (17 intervention and 28 control). See Additional file 1 for a detailed overview of recruitment at each cancer centre. The proportion of eligible participants after screening was higher than anticipated. We estimated 30% of patients screened would score an eligible FCR4 score; however, 42% of participants screened from intervention centres and 55% of participants screened from control centres reported an eligible FCR4 score. Additionally, the interclass correlations (ICC) in FCR4 screening scores were found to be effectively zero (9.8 × 10^−15^), indicating no differences due to clustering in FCR4 screening scores between centres.

Original recruitment targets of 65 in each experimental group were not met. Challenges with recruitment were experienced throughout the study including the national COVID-19 lockdown. A key barrier to recruitment was identifying efficient recruitment pathways. There was significant variability in follow-up practices between participating cancer centres. Centres varied in terms of their follow-up timeline, as well as the size, frequency (regular vs. when needed), and focus (required vs. optional; follow-up only vs. mixed; according to treatment type) of follow-up clinics. Hence, we were required to tailor recruitment approaches to each centre and often had to compromise on efficiency to ensure we could access the appropriate patient population. For example, at one centre, we opted to recruit at a large weekly clinic where there was a mixture of ongoing treatment and follow-up appointments (i.e. many ineligible patients) because dedicated follow-up clinics were only conducted on an ad hoc basis. Administrative capacity was also an issue at one centre whereby staff could not send study information to patients prior to clinics, meaning patients could not be recruited when they attended the clinics. Instead, they were given information and asked to return signed consent forms to the research team if interested. Most patients did not return consent forms and following-up patients added additional administrative pressures for research staff.

**Fig. 1:**
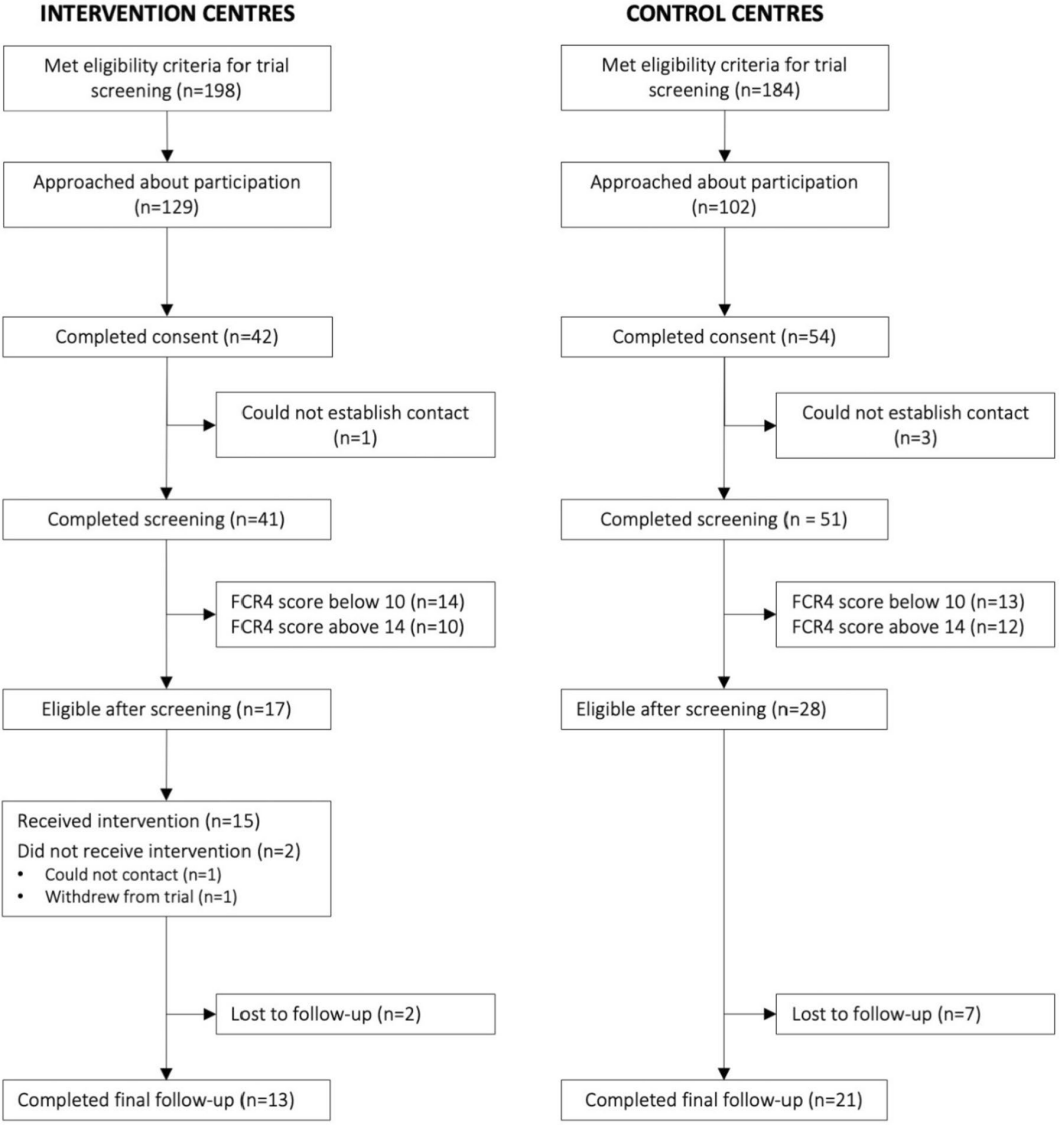
Participant flow through the Mini-AFTERc pilot study, following CONSORT

Of the 17 participants recruited at intervention centres, 15 successfully received the Mini-AFTERc intervention from a SBCN (Fig. 1). One participant withdrew from the trial before receiving the intervention and another participant could not be contacted to arrange the intervention telephone call. Participants were considered to have completed follow-up if they returned a completed FCR4 questionnaire at the final follow-up (3 months). Nine participants (20.93% of the total sample) did not return a final FCR4 questionnaire meaning that the follow-up retention rate across the entire sample was 79.07% (13 intervention and 21 control). There was no difference in retention between intervention and control groups (χ^2^ (1) = 0.803, *p* = 0.37).

All 45 participants who enrolled in the trial, including those who withdrew or were lost to follow-up, were invited to take part in the post-study semi-structured interview via a letter that was sent at the end of their participation. Of these, 10 successfully participated in an interview (1 intervention and 9 control). A second intervention patient was interviewed; however, technical issues with the recording equipment resulted in the loss of this interview.

Data from this pilot study allowed for the estimation of a required sample size required for a full RCT of the Mini-AFTERc intervention. We estimate that 152 (76 intervention and 76 control) completing follow-ups would be required to detect a moderate to large effect size (0.67).

#### Collection of patient outcome measures

Missing data analysis found that 21% of follow-up questionnaire items were missing across the entire sample (intervention and control). Twenty-five participants (58%) had a complete follow-up dataset (i.e. no missing questionnaire items across all 3 follow-up assessments). Little’s MCAR test was not significant (*χ* ^2^ (602) = 370.62, *p* = 1.00) indicating there were no patterns associated with the missing data. Baseline data were not included in the missing data analysis as less than 2% of baseline questionnaire items were missing, the majority of which were attributed to one patient.

Psychometric evaluation of the FCR4 measure was favourable as indicated by the internal consistency coefficient of 0.91 and the average item-scale covariation of 0.66 (Table 2). See Additional file 2 for the results of statistical assumption checks.

**Table 2:** Adjusted FCR4 and single item EQ-5D means, 95%CIs and* p* levels

		**Control**			**Intervention**			
		*Mean*^a^	*95%CI*		*Mean*^a^	*95%CI*		
			*Low*	*High*		*Low*	*High*	*p*
*Measure*	*Time point*							
FCR4	Baseline	11.73	10.87	12.59	11.91	10.69	13.13	0.85
	2 weeks	12.37	11.45	13.29	10.47	9.10	11.83	0.01
	4 weeks	11.52	10.58	12.45	10.65	9.28	12.02	0.20
	12 weeks	12.04	11.10	12.99	10.31	9.02	11.60	0.02
EQ-5D^b^	Baseline	2.04	1.70	2.37	2.07	1.60	2.54	0.92
	12 weeks	2.12	1.75	2.49	1.60	1.11	2.09	0.03

^a^Adjusted for age in years

^b^Anxiety and depression item only

**Fig. 2:**
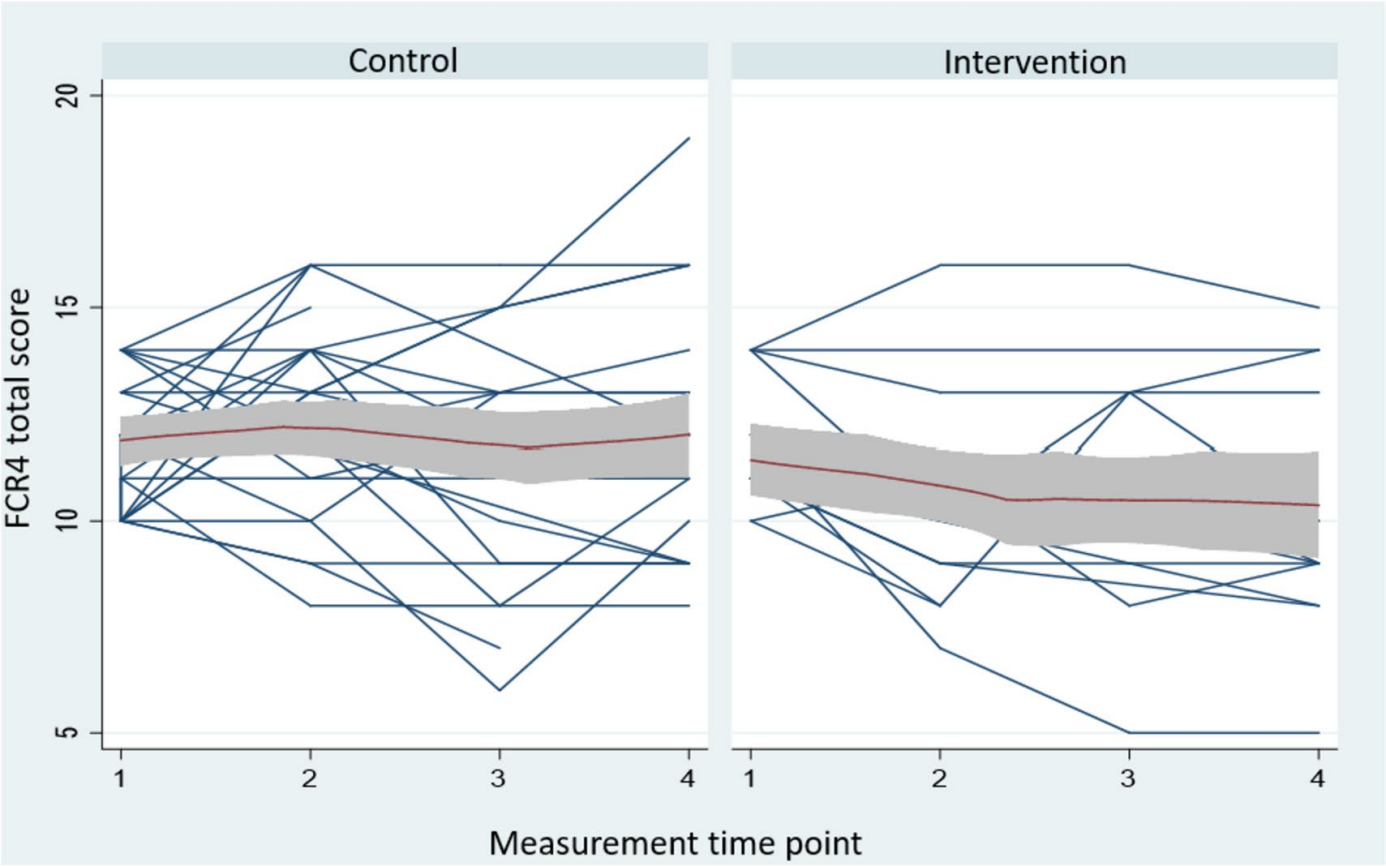
Participant trajectories (blue) clustered by group (control and intervention) with polynomial fitted line (red) and 95%CI (shaded). Measurement time points: 1 = baseline, 2 = 2 weeks, 3 = 4 weeks, 4 = 12 weeks

A mixed linear growth model for the FCR4 assessments, using baseline and follow-up data, showed an overall significant time by group interaction effect (*p* = 0.038, two-sided). Plotting the adjusted means (Fig. 2) identified a significant comparison in favour of the Mini-AFTERc between intervention and control participant self-reports were confirmed at 2 weeks and 12 weeks following baseline (*p* < 0.03 for both). The effect size (Cohen’s *d*) for the 12-week follow-up assessment was 0.48.

#### Evaluation of methodological components

Comprehensive methodological evaluation according to the ADePT framework found most components of the study worked well together (see Table 3). The SBCN training programme was successful and promptly delivered with positive feedback from SBCNs. Patient screening, consent, and follow-up protocols were successful and retention rates were excellent. Although recruitment presented challenges and overall targets were not met, all centres recruited successfully.

**Table 3:** Evaluation of methodological components of the Mini-AFTERc pilot study

**Methodological issues**	**Findings**	**Evidence**
1. Did the feasibility/pilot study allow a sample size calculation for the main trial?	We estimate a total sample size of 152 completing follow-up would be required for a main trial.	A sample size calculation (n=152 reaching follow-up) has been performed from the estimated effect size of 0.67. This would still be achievable with four cancer centres if they can identify approximately 400 patients each (Total eligible = 1689 patients).
2. What factors influenced eligibility and what proportion of those approached were eligible?	FCR4 cut-offs were the key factor in eligibility. A greater than anticipated proportion of patient screened met FCR4 screening criteria (25% anticipated; 49% actual). One third of patients who were ineligible after screening scored just above the FCR4 cut-off (score of 15).	45 of 92 patients (49%) who completed screening were eligible based on FCR4 cut-offs. 32% or 15 of the 47 ineligible patient (FCR score out with 10-14) scored just above the cut-off (15 or 16). Increasing the cut-off to include this group is theoretically justifiable and may boost recruitment potential significantly.
3. Was recruitment successful?	Overall recruitment targets of 50 patients in each study group was not achieved. Recruitment to control group was more successful than to intervention group. Recruitment was impacted by: • Variability in participating cancer centres’ follow-up procedures. • Administrative restrictions in one centre. • Systemic issues with SBCNs finding protected time to practice the intervention. • National lockdown due to the Covid-19 pandemic.	28 of 51 patients screened at control centres were eligible and successfully recruited into the control group (56% of expected control sample of 50 patients). 17 of 41 patients screened at intervention centres were successfully recruited into the intervention group (34% of expected intervention sample of 50 patients). 49% of those screened were eligible (45 of 92 patients)
4. Did eligible participants consent?	All participants who were eligible after screening agreed to participate.	100% of those screened as eligible (45 patients; 49.4% of total screened) agreed to participate in the study.
5. Were participants successfully randomized and did randomization yield equality in groups?	Not applicable.	Not applicable.
6. Were blinding procedures adequate?	Not applicable.	Not applicable.
7. Did participants adhere to the intervention?	Patient adherence to the intervention was not assessed because Mini-AFTERc does not require adherence to specific criteria or conditions. A fidelity of intervention implementation measure has been developed to assess SBCN adherence to the Mini-AFTERc training manual.	The fidelity of implementation measure has preliminary evidence of good reliability: Brandt, N. G., McHale, C. T., & Humphris, G. M. (2020). Development and Testing of a Novel Measure to Assess Fidelity of Implementation: Example of the Mini-AFTERc Intervention. *Frontiers in psychology*, *11*, 601813. 10.3389/fpsyg.2020.601813
8. Was the intervention acceptable to the participants?	Both patients and nurses found the Mini-AFTERc intervention to be valuable and acceptable.	13 of 15 intervention patient participants completed CARE and modified-MISS questionnaires following the intervention discussion. Mean scores for all 10 care questions were between 4 and 5 (very good and excellent). Mean scores for modified MISS questions: patients neither agreed or disagreed with statements that the nurse had relieved their fear about the cancer returning and that they felt much better about the chances of the cancer not coming back. Patients disagreed with the statement that talking to the nurse had not helped at all. Patient interviews (9/10 control patient): Patients perceived a general lack of structured psychosocial support from the cancer centre following the end of primary treatment. They thought that it was important and acceptable to provide FCR intervention for those who needed it. SBCN interviews: SBCN valued the structured approach of the Mini-AFTERc intervention, it helped them with discussion time management, rose awareness of FCR, increased their confidence in talking about FCR. SBCN thought Mini-AFTERc was a more strcutred approach to the FCR discussion they had with patients regularly
9. Was it possible to calculate intervention costs and duration?	The cost of intervention implementation was not assessed but it was designed to be integrated into current cancer care practice by making use of existing staff and resources. Each intervention discussion is structured to last a maximum of 30 minutes. Overall time would be determined by how many patients receive it in practice and/or whether it is only offered to certain patients.	SBCN interviews: Some SBCN did raise concerns about finding protected time within the current workload to conduct the Mini-AFTERc intervention.
10. Were outcome assessments completed?	Patient outcome assessments were well completed.	80% of follow-up questionnaire items were successfully competed by patient participants. Missing follow-up items were ‘missing complete at random’ and there were no patterns to data missingness; χ^2^ (602) = 370.62, *p* = 1.00).
11. Were outcomes measured those that were the most appropriate outcomes?	FCR4 questionnaires, which were the primary patient outcome measure, were well completed by patients. The mobile phone application was not utilised very often, primarily due to Operating System restrictions (Apple iOS version not available). All patient were happy to complete pencil and paper version of questionnaires.	Missing data on completion of the FCR4 items was almost non-existent. That is, of the patients who attempted completion of the 4 items it was found that all 4 items were responded to without items being missed out. The Raykov's factor reliability coefficient for FCR4 was 0.913 (equivalent to Cronbach’s alpha). Confirmatory factor analysis of FCR4 showed a ‘very close fit’ to a unidimensional latent variable. Chi square equalled a low value of misfit (p>.3), indicating exceptional measurement clarity. FCR4 scoring had little to no floor or ceiling effect. One percent scored at the lowest possible rating on the total score and two of the patients (2%) scored at the highest but one value on the measure.
12. Was retention to the study good?	Retention rate, defined as retuning the final (12 week) FCR4 follow-up questionnaire, was almost 80%. Two patients dropped out before receiving the intervention. Both were recruited just prior to the national Covid-19 lockdown, when there was a lot of uncertainty about health service provision.	9 out of 45 patients did were lost to follow-up (20.1%).
13. Were the logistics of running a multicentre trial assessed?	Recruitment approaches varied by centre due to differences in follow-up practices, which influenced the success of recruitment at each centre. All centres did recruit patients. Administrative and organisational issues challenged recruitment (see response to Q3). For this study, two research staff (supported by PIs) were able to co-ordinate and manage research activities at the four centres effectively.	Recruitment was less efficient at the centre where administrative support from the centre was restricted. The centre where recruitment was conducted entirely via telephone was as successful as other centres were recruitment was conducted in person.
14. Did all components of the protocol work together?	Training, screening, consent and follow-up protocols were successful, and all worked well together. Intervention implementation was successful conducted by the SBCNs. Patient interviews were successfully conducted but were heavily skewed towards control patients. Interviews were conducted during the first Covid-19 national lockdown in the UK (May-June 2020). Recruitment protocols worked and patients were recruited, however recruitment was challenged by various factors, which reduced the efficiency of recruitment at some centres.	The SBCN training programme was successful and promptly delivered with positive feedback from SBCNs. 96% of patients were screening after providing informed consent and 100% agreed to participation if eligible after screening. Retention rate across the sample was 80%. Required number of interviews were met with control patients (35% actual, 25% expected) but not with intervention patients (11 %actual, 25% expected). All patient who were due to receive the Mini-AFTERc intervention did within two weeks of recruitment.

## Discussion

This pilot study aimed to investigate the acceptability and feasibility of assessing the Mini-AFTERc intervention in a controlled trial format and its implementation into clinical practice. A comprehensive Mini-AFTERc training programme was developed and successfully delivered to SBCNs. We found that the Mini-AFTERc intervention was acceptable to both SBCNs and patients. SBCNs were able to successfully deliver the intervention but expressed some doubts about the feasibility of routinely implementing the Mini-AFTERc intervention at scale, specifically how they could fit the delivery of Mini-AFTERc within their already significant workload. Recruitment was challenged by the variability in cancer centres’ follow-up procedures and was prematurely halted due to the COVID-19 pandemic. Nevertheless, it was supported by high levels of participation after consent and retention in both intervention and control centres. Patient outcome measures were completed well and preliminary evidence for the efficacy of the Mini-AFTERc intervention to reduce FCR at 12 weeks post-intervention was identified.

As part of this research, we developed a comprehensive nurse-led Mini-AFTERc training programme, combining education and knowledge acquisition with role-play and constructive feedback. Our training approach is substantiated by previous work evaluating approaches to communication training in cancer care, concluding that learner-focused training programmes concentrating on developing theoretical understanding and providing practical rehearsal and peer feedback were very effective [30, 31]. As the aim of this pilot study was to *develop* the Mini-AFTERc training programme, we did not conduct a formal training evaluation; however, all training sessions were delivered successfully and efficiently, and all SBCNs expressed that they found the training valuable and useful. A key objective for a planned future RCT of the Mini-AFTERc intervention should be to conduct a full training evaluation, in terms of satisfaction, knowledge acquisition, and transfer of learning into practice. A logic model approach to training programme design and evaluation is recommended as it ensures a thorough and rigorous evaluation [32].

Assessments of intervention acceptability should examine the anticipated or experienced cognitive (e.g. coherence, effectiveness) and emotional (e.g. affective attitude, burden) response of those receiving and delivering the intervention [33]. Measurements of patient satisfaction following Mini-AFTERc intervention discussions found that participants perceived the emotional and relational empathy of SBCNs to be excellent (CARE questionnaire). Participants were more neutral about perceived effectiveness (MISS questionnaire); however, measures were completed immediately following the intervention and may have been too soon for them to appraise its benefits. Acceptability was also discussed during post-study interviews; however, only two participants who received the intervention came forward for the interview and, unfortunately, one of these interviews did not audio record correctly. Therefore, most of the qualitative data came from patients who did not actually receive the intervention. Although they did not take part in the intervention, control patients understood the purpose of the Mini-AFTERc intervention (after explanation) and supported its implementation for those who may need it. For many of these patients, discussion about the Mini-AFTERc intervention also triggered a broader discussion about a perceived lack of psychological and lifestyle support more generally from their cancer centre following primary treatment. This reflects a longstanding narrative of unmet needs following cancer treatment [34] and reinforces a need for more structured psychosocial interventions (such as Mini-AFTERc) for cancer survivors.

Participants were relaxed about potential randomisation procedures in a future trial and some expression of receipt of the intervention in control participants would be considered advantageous following the final follow-up assessment to maintain fairness.

Recruitment was our primary focus when evaluating the feasibility of this pilot study. Although we did not achieve our intended recruitment targets, we were still able to recruit successfully at all centres. Significant variability in cancer centre follow-up procedures, as well as their ability to support the administration associated with recruitment, impacted our ability to recruit *efficiently* to the study. In keeping with the ADePT process [25], our evaluation focused on the generation and appraisal of possible solutions to the issues we experienced with recruitment. Increasing the capacity to recruit by employing more dedicated researchers would be an obvious solution. However, our recruitment issues were more related to a lack of suitable clinics to attend rather than a lack of researchers to attend them. Follow-up appointments are dependent on many factors (such as treatment type and cancer severity) and many are often conducted as needed, rather than in a regular clinic. As such, it would be advantageous to have research personnel continually present in the centre and able to recruit opportunistically. Enabling the SBCNs to recruit patients or the deployment of dedicated clinical research nurses could facilitate more efficient patient recruitment in these ad-hoc environments.

Following our analysis of patient screening and outcome data, changes to the study design and methodology could be justified to facilitate recruitment. First, fifteen patients were found to score 15 or 16 on the FCR4 screening questionnaire, falling just a couple of scale points outside of the upper FCR4 cut-off of 14. Our psychometric evaluation of the FCR4 data suggests that the difference between the upper cut-off of 14 and a score of 16 may be arbitrary and that increasing FCR4 cut-off to 16 is justifiable. Based on our data, this would increase the patient eligibility rate by approximately 16% which would be significant given the very high rate of consent to participate after screening. Secondly, it was found that there was no difference in patient FCR4 screening scores between cancer centres highlighting that any centres that are underperforming in terms of recruitment could be supported by centres that are meeting or exceeding recruitment targets. This was not possible during this pilot study as centres were set as either intervention or control recruiters. A future study may employ randomisation stratification by centre, thereby ensuring a balanced allocation of participants to both control and intervention groups.

### Strengths and limitations

This study is the first to demonstrate that a brief telephone intervention aimed at preventing FCR development is acceptable to patients and can be feasibly delivered by existing members of the cancer care team, which is advantageous in the current environment of limited health service funding and remote consultations. Our patient interview sample was heavily skewed towards patients from control centres as few patients from intervention centres came forward for interview. This meant that we were unable to obtain representative more in-depth qualitative views from patients who received the Mini-AFTERc intervention. It is unclear why so few intervention patients came forward for interviews. Our interviews were conducted during the first few weeks of the initial COVID-19 lockdown in Scotland. Uncertainty about risk and support as cancer services reduced during this time may have impacted participants’ willingness to discuss FCR with researchers. Although retention and rates of questionnaire completion were good, the overall sample was small limiting the power of quantitative analysis. Nevertheless, the data were sufficient for our purposes (e.g. calculate projected sample sizes for an RCT) and we were able to show evidence of intervention effectiveness, although this was not a primary objective of this pilot work. Readers are alerted to the non-randomisation of centres in this pilot which may have introduced bias in estimating the benefit of the intervention.

Finally, we focus on researchers who are interested to promote the testing of new brief FCR interventions to concentrate particularly on:

First, the design and flexible training to meet the needs of the staff present who are willing to introduce the intervention such as Mini-AFTERc. The training programme might benefit from a logic model design.

Second, to concentrate specifically on the administrative process of patients being guided through the treatment and follow-up services to enable clear points of contact with key-staff and clinic routines. An awareness of the variability of each centre introduced into the study will increase the efficiency of recruitment.

Third, consider the inclusion in the study design of dedicated external research personnel (clinical research nurses) to identify eligible follow-up patients not only for recruitment, consenting, and entry into the study but also to support follow-up.

More generally the study demonstrated that a key point, soon after the conclusion of active treatment for breast cancer, presents itself for identifying and satisfying the needs of patients. The Mini-AFTERc intervention and its initial implementation experiment have shown some promise to assist the psychological health of women beyond the diagnosis and treatment stages of this common disease.

## Conclusion

This controlled pilot study of the Mini-AFTERc intervention for fear of cancer recurrence found that Mini-AFTERc was acceptable to both patients receiving the intervention and SBCNs delivering it. The intervention was implemented by SBCNs successfully; however, they perceived challenges with implementation of Mini-AFTERc on a larger scale due to workload. Methodologically, all aspects of the study worked well. Although recruitment was impacted by variability in centre follow-up practices and the Covid-19 lockdown, all centres recruited successfully. The study provided invaluable information about the potential challenges and solutions for testing the Mini-AFTERc intervention more widely where limiting high FCR levels is an important goal following recovery from primary breast cancer treatment.

### Supplementary Information

**Additional file 1.** MiniAFTERc recruitment and retention per site.

**Additional file 2.** FCR4 assumption tests.

## Data Availability

On request from the corresponding author.
