# Supplementary material for: Mini-AFTERc: a controlled pilot trial of a nurse-led psychological intervention for fear of breast cancer recurrence
Source: Pilot Feasibility Stud. 2024 Jan 8;10:3. doi: 10.1186/s40814-023-01431-x (PMC10773079; doi:10.1186/s40814-023-01431-x)
Supplement: Supplementary file 1 — Additional file 1. MiniAFTERc recruitment and retention per site. [file 40814_2023_1431_MOESM1_ESM.docx]

**Supplementary information 1: Eligibility and recruitment summary by centre**

| **Centre ^1^** | **Potentially eligible ^2^** | **Approached about study ^3^** | **Completed consent ^4^** | **Completed screening ^5^** | **Eligible and participated ^6^** | **Completed follow-up ^7^** |
| --- | --- | --- | --- | --- | --- | --- |
| S1 | 103 | 64 | 30 | 30 | 16 | 12 |
| S2 | 133 | 72 | 21 | 20 | 8 | 7^*^ |
| S3 | 81 | 38 | 24 | 21 | 12 | 9 |
| S4 | 65 | 57 | 21 | 21 | 9 | 6^*^ |
|  |  |  |  |  |  |  |
| **Total** | **382** | **231** | **96** | **92** | **45** | **34** |

Notes

^1^ S1 and S3 were control centres. S2 and S4 were intervention centres.

^2^ All patient who met the inclusion criteria for the study, *excluding FCR4*.

- S1, S3, S4: Everyone who was sent a letter by the centre. Patients were not sent a letter unless they met the inclusion criteria. Clinical staff determined patient eligibility using patient records.
- S2: Numbers based on reports from clinical staff as to how many patients in each clinic met the inclusion criteria, and subsequently estimated averages. Not all patients attending clinics met the inclusion criteria, therefore it was at the discretion of the clinician as to who did meet the criteria. Patients could not be sent letters prior to appointments due to administrative issues.

^3^ All patients who were directly approached by a clinician or researcher (at clinics or over the phone) about participating in the study.

- S1 & S4: Patients asked, by nurses, if they would like to speak to the research at the end of their appointment. All had received study letters before the appointment.
- S2: Patient brought to see researcher by the oncologist at the end of their appointment.
- S3: Patients who verbally consented (during a treatment follow-up call) to their contact details being passed onto researchers by the nurses. All had received study letters before the call.

^4^ All patients who completed a consent form, either in the clinic with the researcher or returned it through the mail.

^5^ All patients who had returned consent and successfully completed the screening questionnaires (FC4 and demographics) either in the clinic or over the phone.

^6^ All patients who were within the FCR4 cut-off limits (10-14) in their screening questionnaire. All patients found to be eligible agreed to participate.

^7^ All participants who returned the final follow-up questionnaire, they may have or have not completed the intermediate follow-up questionnaires.

^*^ One patient in S4 withdrew prior to receiving the intervention telephone call and one patient in S2 could not be contacted prior to receiving the intervention telephone call, therefore they were not followed up. This occurred during the period where the country was entering lockdown.
