# Supplementary material for: Mini-AFTERc: a controlled pilot trial of a nurse-led psychological intervention for fear of breast cancer recurrence
Source: Pilot Feasibility Stud. 2024 Jan 8;10:3. doi: 10.1186/s40814-023-01431-x (PMC10773079; doi:10.1186/s40814-023-01431-x)
Supplement: Supplementary file 2 — Additional file 2. FCR4 assumption tests. [file 40814_2023_1431_MOESM2_ESM.docx]

Supplementary file

Psychometric details of FCR4 scale

Item | Obs Mean Std. Dev. Min Max

-------------+---------------------------------------------------------

fcr1 | 91 3.054945 .9233109 1 5

fcr2 | 91 2.945055 .8738502 1 5

fcr3 | 91 2.989011 .98313 1 5

fcr4 | 91 2.516484 1.03669 1 4

fcrt | 91 11.50549 3.403833 4 19

average

item-test item-rest interitem

Item | Obs Sign correlation correlation covariance alpha

-------------+-----------------------------------------------------------------

fcr1 | 91 + 0.8891 0.8035 .6742369 0.8858

fcr2 | 91 + 0.8948 0.8193 .6888482 0.8823

fcr3 | 91 + 0.9114 0.8343 .6269434 0.8745

fcr4 | 91 + 0.8730 0.7589 .6528694 0.9040

-------------+-----------------------------------------------------------------

Test scale | .6607245 **0.9124**

-------------------------------------------------------------------------------

Interitem covariances (obs=91 in all pairs)

fcr1 fcr2 fcr3 fcr4

fcr1 0.8525

fcr2 0.5808 0.7636

fcr3 0.6895 0.6883 0.9665

fcr4 0.6713 0.6287 0.7057 1.0747

Cut-

fcrt | Freq. Percent Cum. Off N %

--------+-----------------------------------

4 | 1 1.10 1.10

5 | 2 2.20 3.30

6 | 2 2.20 5.49

7 | 7 7.69 13.19

8 | 6 6.59 19.78

9 | 9 9.89 29.67

**10 | 10 10.99 40.66**

**11 | 11 12.09 52.75**

**12 | 11 12.09 64.84**

**13 | 3 3.30 68.13**

**14 | 9 9.89 78.02 10-14 44 48.35**

15 | 5 5.49 83.52 10-15 49 53.85

16 | 10 10.99 94.51 10-16 59 64.84

17 | 2 2.20 96.70

18 | 1 1.10 97.80

19 | 2 2.20 100.00

--------+-----------------------------------

Total | 91 100.00

No appreciable floor or ceiling effects. That is the full range of scores are being used with no clustering of cases at low or high limits of scale.

. sktest fcrt

Skewness/Kurtosis tests for Normality

------ joint ------

Variable | Obs Pr(Skewness) Pr(Kurtosis) adj chi2(2) Prob>chi2

-------------+---------------------------------------------------------------

fcrt | 91 0.6914 0.1007 2.94 0.2302

The probability of the FCR4 total showing a non-significant difference from kurtosis of a normal distribution is 0.1007 (that is greater than 10%). Skewness is almost 70% non-significantly different from a normal distribution. The combined skewness and kurtosis is also non-significant (23%).

. summarize fcrt, detail

fcrt

-------------------------------------------------------------

Percentiles Smallest

1% 4 4

5% 6 5

10% 7 5 Obs 91

25% 9 6 Sum of Wgt. 91

50% 11 Mean 11.50549

Largest Std. Dev. 3.403833

75% 14 17

90% 16 18 Variance 11.58608

95% 17 19 Skewness .0954554

99% 19 19 Kurtosis 2.334342
